# Tobacco Cessation and Prevention Interventions for Sexual and/or Gender Minority-Identified People and the Theories That Underpin Them: A Scoping Review

**DOI:** 10.1093/ntr/ntad018

**Published:** 2023-01-31

**Authors:** Julia McQuoid, Arturo Durazo, Evan Mooney, Jaimee L Heffner, Andy S L Tan, Amanda Y Kong, Shari Clifton, Elizabeth Horn

**Affiliations:** Department of Family and Preventive Medicine and TSET Health Promotion Research Center, University of Oklahoma Health Sciences Center, 655 Research Parkway, Suite 400, Oklahoma City, OK, USA; Health Sciences Research Institute and Nicotine & Cannabis Policy Center, University of California, Merced, Merced, CA, USA; Department of Family and Preventive Medicine, University of Oklahoma Health Sciences Center, Oklahoma City, OK, USA; Cancer Prevention Program, Public Health Sciences Division, Fred Hutchinson Cancer Center, Seattle, WA, USA; Annenberg School for Communication, University of Pennsylvania, Philadelphia, PA, USA; Department of Family and Preventive Medicine and TSET Health Promotion Research Center, University of Oklahoma Health Sciences Center, 655 Research Parkway, Suite 400, Oklahoma City, OK, USA; Health Sciences Library and Information Management, Graduate College, Robert M. Bird Health Sciences Library, University of Oklahoma Health Sciences Center, Oklahoma City, OK, USA; Freedom Oklahoma, Oklahoma City, OK, USA

## Abstract

**Introduction:**

This scoping review takes stock of the social and behavior change theories that have underpinned tobacco interventions tailored to sexual and/or gender minority (SGM) people and reflects on the need to target contextually based drivers of SGM tobacco use inequities.

**Aims and Methods:**

Data sources were Medline (Ovid), Scopus, PubMed, and Google Scholar (January 01, 1946 to October 27, 2022). Peer-reviewed publications in English from anywhere in the world describing SGM-tailored tobacco cessation and/or prevention interventions were independently identified by a librarian and screened by the first and third authors. Three hundred and sixty-seven articles were extracted; an additional two were found by hand searching. A total of 369 articles were assessed for eligibility. Exclusion criteria were: Not an intervention, review article, not SGM-tailored, or tobacco-focused. We documented the intervention name, intervention components, theoretical frameworks cited in reference to intervention design and/or implementation, and evaluation outcomes. All authors provided input on theoretical framework categorization.

**Results:**

We identified 22 publications corresponding to 15 unique interventions. Individual-level behavior change theories (ie, those focusing on within-person behavior change processes) were the most prominent. Among these, the Transtheoretical Model was the most frequently utilized, while Social Inoculation Theory, Theory of Reasoned Action, and Theory of Psychological Reactance were also employed. A minority of interventions referenced frameworks that more explicitly engaged with SGM people’s social contexts, namely, Theory of Diffusion of Innovations and Minority Stress Model.

**Conclusions:**

Future SGM-tailored tobacco interventions should leverage both the strengths of individual-level behavior change theories and those of frameworks that understand tobacco use inequities as indivisible from place, context, and policy.

**Implications:**

This scoping review describes the theoretical underpinnings of sexual and/or gender minority (SGM)-tailored tobacco interventions published in the peer-review literature in English. It reflects on the need for greater utilization of social and behavior change theoretical frameworks that can engage with unique drivers of SGM tobacco use and barriers to cessation.

## Introduction

Persistently high tobacco use rates among sexual and/or gender minority (SGM) groups^1–3^ have roots in the social, political, and physical environments that SGM people live in and the ways in which tobacco use is linked to interacting with and negotiating these environments as a socially minoritized person.^4–9^ For example, studies from predominantly English-speaking countries have attributed tobacco use among SGM individuals to cope with experiences of social minority stress, including internalized homophobia, fear of rejection, harassment, and violence.^4,5,10–17^ Tobacco use has also been described by SGM individuals as a way to symbolically resist oppression by engaging in a “counterculture” practice.^18,19^ The tobacco industry capitalizes on the motives that many SGM people report for using tobacco and has long promoted tobacco use among SGM groups with targeted tobacco marketing campaigns.^20–24^ Consequently, pro-tobacco norms are prevalent within SGM social networks, and tobacco use is highly visible within SGM-oriented media and spaces.^19,25–29^ Promoting health equity for SGM groups through tobacco-free living must be done in a way that can address these unique drivers of use at the individual, network, community, and ecological levels, as well as the barriers that SGM people have to access tobacco cessation assistance^30,31^ and the preference SGM people have expressed for tailored and culturally relevant intervention approaches.^32–35^

There has been an increase in tailored tobacco cessation and prevention interventions for SGM people in the past decade,^36–38^ although early tobacco control efforts for SGM communities began at least in the early 1990s.^39^ These efforts have been concentrated in the United States; as of 2017, 79% of SGM-tailored tobacco cessation interventions originated in the United States.^37^ The modest but growing body of scholarly evidence on SGM-tailored tobacco interventions presents an opportunity to take stock of the social and behavior change theories that have underpinned these intervention efforts thus far. It also provides an opportunity to consider how theories that have been employed in other health behavior change fields may be leveraged to further engage with drivers of SGM tobacco use that are perpetuated within person-environment interactions.

Following Glanz et al,^40,41^ social and behavior change theories each offer “a systematic way of understanding events, behaviors and/or situations”.^40^ They consist of “a set of interrelated concepts, definitions, and propositions that explain or predict events or situations by specifying relations among variables”.^40^ Increasing evidence suggests that public health and health-promoting interventions that are informed by social and behavior change theories are more effective than those lacking an explicit theoretical base.^40,42–44^ Moreover, the particular set of factors that a social and/or behavior change theory relies upon to explain or predict health behavior (eg, affect, knowledge, attitudes, social networks, culture, and geography) also appears to influence the effectiveness of the interventions informed by that theory.^40,45,46^ For example, interventions that focus on individual-level processes of behavior change will influence different factors related to smoking than interventions that also account for the social and physical environments of smoking behavior and smoking cessation.

Although prior reviews of SGM-tailored tobacco interventions exist,^36–38^ none have specifically examined the social and behavior change theories that have underpinned these interventions. To address this gap in the literature, we conducted a scoping review with the objective of assessing the theoretical influences driving the current direction of the scientific field of SGM-tailored tobacco intervention design and implementation. We limit our scoping review to publications that have gone through the peer-review process, which is meant to improve the scientific accuracy and quality of published reports.^47^

## Methods

We conducted a scoping review of peer-reviewed articles published in English from anywhere in the world that described SGM-tailored tobacco cessation and prevention interventions and identified any theoretical frameworks referenced therein. A comprehensive Ovid MEDLINE search was performed by a health sciences librarian (SC) for the time period of January 1, 1946 (database inception) to October 27, 2022. The full electronic search strategy for Ovid MEDLINE is provided in Supplementary Appendix A. This yielded 367 unique records. Two (2) additional unique records were identified by the first and third author with forward and backward citation tracking (ie, hand searching) and supplemental searches in Scopus, PubMed, and Google Scholar with the keywords “sexual and gender minorities”, OR “LGB,*” AND “smoking cessation”, OR “tobacco intervention”.

**Figure 1.**
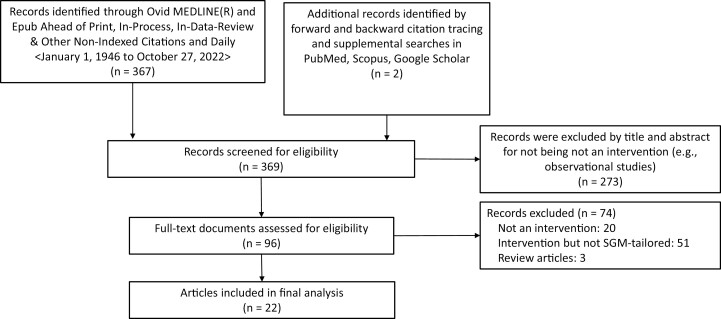
Identification and selection.

Figure 1, below, displays the article identification and selection process that was followed by the first and third authors. Inclusion criteria were any peer-reviewed work published in the English language (January 1, 1946 to October 27, 2022) describing or reporting the outcomes of an intervention tailored to reduce, prevent, or stop tobacco use among SGM populations. By “interventions” we mean behavioral interventions, pharmacotherapy, clinical approaches, communication and media campaigns, public policy, and combinations thereof. By “tobacco”, we mean any nicotine-containing product other than U.S. Food and Drug Administration-approved medications for tobacco cessation (eg, nicotine replacement therapy). By SGM-tailored, we mean enhanced and/or informed intervention approaches for SGM populations that specifically take into account characteristics shared by SGM people. We endorse the term “tailored” over “targeted” because of the connotations of violence in the latter term.^48^ Exclusion criteria were: (1) literature reviews or other work that does not report primary research on an SGM-tailored tobacco intervention, (2) non-peer-reviewed work, and (3) formative work to inform future SGM-tailored tobacco intervention design. Ultimately, we identified 22 articles for inclusion in the final analysis that described or reported on outcomes of a total of 15 unique SGM-tailored tobacco interventions.

The first author read the articles included for final analysis (*n* = 22) in full and recorded the following dimensions for each article:

- Intervention name (or concise intervention description if not named).
- Main intervention delivery components (eg, group counseling, 7-week program, began with an educational focus, and shifted to a social support focus in later weeks).
- Any social and/or behavior change theories referenced in the article (eg, Diffusion of Innovations).
- Evaluation components (eg, sample size, use of comparison or control group, primary outcome measures).

All authors provided input on decisions regarding how to describe and categorize the theories underpinning each intervention.

### Findings

Table 1 summarizes the social and behavior change theoretical frameworks underpinning peer-review published SGM-tailored tobacco interventions. Frameworks are grouped by the theories that informed them and listed in order of most to least cited. Five interventions were not linked to a specific theory in their corresponding publication(s), or, they mentioned concepts that were likely related to specific theoretical underpinnings, but did not make this explicit (eg, “psychosocial treatment”; “cognitive behavioral program”).^49–51^ Several interventions were linked to multiple theories. Multiple articles associated with the same intervention are included in the table as cases were found where all the theories that informed the intervention were not listed exhaustively in any one publication associated with that intervention.

**Table 1.** Theoretical Frameworks Informing Sexual and/or Gender Minority (SGM)-tailored Tobacco Interventions Published in the Peer-reviewed Literature (*n* = 15 Unique Interventions; January 1, 1946 to October 27, 2022)

Social and/or behavioral theory	# Interventions	Intervention name(s) and main intended outcome	Corresponding peer-reviewed publication(s)
**Unspecified theoretical framework**			
No explicit theoretical framework	5	Queer Quit *-Cigarette smoking cessation* Project Exhale *-Cigarette smoking cessation* Freedom from Smoking^a^ *-Cigarette smoking cessation* Location-based media campaign for lesbian and bisexual women *-Promote cigarette smoking cessation and awareness of state quitline* Smoking cessation for gay men *-Cigarette smoking cessation*	Dickson**-**Spillmann et al. 2014 Matthews, Conrad, et al. 2013 Matthews, Li et al. 2013 Caldwell et al. 2022 Harding et al. 2004
**Individual-level theories of behavior**			
Transtheoretical Model	3	The Last Drag *-Cigarette smoking cessation* Courage to Quit^a^ *-Cigarette smoking cessation* The Put It Out Project *-Cigarette smoking cessation*	Eliason et al. 2012 Walls and Wisneski 2011 Williams et al. 2020 Matthews et al. 2014 Matthews, Steffen et al. 2019 Vogel et al. 2020 Vogel et al. 2019
Theory of Reasoned Action or Theory of Planned Behavior ^b^	3	This Free Life *-Change beliefs about cigarette smoking* This Free Life + local Social Branding *-Increase intentions to quit cigarette smoking* Truth Campaign^a^ *-Increase support for tobacco control policies and anti-tobacco industry sentiments*	Guillory et al. 2021 Crankshaw et al. 2022 Navarro et al. 2019 Hinds et al. 2021 Beckerley et al. 2022 Skurka et al. 2021
Social Inoculation Theory	2	This Free Life *-Change beliefs about cigarette smoking* Truth Campaign^a^ *-Increase support for tobacco control policies and anti-tobacco industry sentiments*	Guillory et al. 2021 Crankshaw et al. 2022 Navarro et al. 2019 Hinds et al. 2021 Skurka et al. 2021
Theory of Psychological Reactance	2	This Free Life *-Change beliefs about cigarette smoking* Truth Campaign^a^ *-Increase support for tobacco control policies and anti-tobacco industry sentiments*	Guillory et al. 2021 Crankshaw et al. 2022 Navarro et al. 2019 Hinds et al. 2021 Skurka et al. 2021
Health Belief Model	1	Courage to Quit^a^ *-Cigarette smoking cessation*	Matthews et al. 2014 Matthews, Steffen et al. 2019
Relational Frame Theory	1	Empowered, Queer, Quitting, and Living (EQQUAL) *-Cigarette smoking cessation*	Heffner et al. 2021
Self-Determination Theory	1	Proactive Outreach Letter^a^ *-Increase uptake of state quitline cigarette smoking cessation services*	Matthews, Breen et al. 2019
**Theories of social context and behavior**			
Diffusion of Innovations Theory	3	CRUSH *-Reduce cigarette smoking prevalence* Break Up *-Reduce cigarette smoking prevalence* This Free Life + local Social Branding *-Increase intentions to quit cigarette smoking*	Fallin et al. 2015 Plant et al. 2017 Beckerley et al. 2022
Minority Stress Model	1	Courage to Quit^a^ *-Cigarette smoking cessation*	Matthews et al. 2014

^a^Indicates an SGM-tailored version of the intervention.

^b^Usually classified together under the Reasoned Action Approach umbrella^52^.

The main intended outcome of each intervention is noted in italics beneath the intervention name. As observed in Table 1, the majority of interventions focused on cigarette smoking cessation, with a minority focusing on beliefs, norms, and/or intentions related to cigarettes. Nicotine-containing products other than cigarettes (eg, e-cigarettes, smokeless tobacco) were not targeted. As an exception, one intervention^53^ aimed to encourage support for tobacco control policies and anti-tobacco industry sentiment rather than focusing on cigarettes specifically.

Individual-level behavior change theories—meaning those that focus on explaining within-person processes of behavior change—were the most prominent. Among these, the most utilized theory was the Transtheoretical Model, cited in the articles describing three interventions.^54–59^ This framework delineates a process in which individuals move through various “stages of readiness” for behavior change, from pre-contemplation to sustained termination of smoking.^60^ SGM-tailored interventions have “stage-matched” their engagement with participants according to where they fall on the theorized behavior change continuum.^56,57^ One intervention cited the health belief model,^58,61^ which purports that a person’s belief in the personal threat of tobacco-related harm, and the extent to which they believe that abstaining from tobacco use will benefit them, will predict tobacco cessation motivation and outcomes.^62^ Another study used relational frame theory,^63^ which underpins acceptance and commitment therapy, to support SGM smoking cessation.^63^ Acceptance and commitment therapy focuses on building psychological flexibility—defined as a willingness to experience uncomfortable thoughts, feelings, and physical sensations without attempting to change them—and having clarity of one’s values to act in a way that is in service of those values.^63,64^ Self-determination Theory,^65^ cited as informing one intervention,^66^ emphasizes the importance of competence, relatedness, and autonomy in the successful achievement of goal-directed behavior, like smoking cessation.

SGM-tailored tobacco interventions have also utilized public education media campaigns that deliver counter-tobacco industry messaging.^53,67–71^ While the publications describing these SGM-tailored interventions may not explicitly cite theory, Hersey et al^72^ argue that the influence of these types of anti-tobacco campaigns on the beliefs, attitudes, and behaviors of those exposed to messaging is best explained by Social Inoculation Theory,^73^ Theory of Reasoned Action,^74^ and Theory of Psychological Reactance.^75^ Social Inoculation Theory explains how exposing people to small “doses” of tobacco industry marketing alongside delegitimizing arguments can protect them from being persuaded by subsequent exposure to tobacco marketing. The Theory of Reasoned Action focuses on attitudes and intentions toward a behavior; if anti-tobacco industry messaging can shift beliefs about the tobacco industry, it may also shift attitudes about and intentions to use tobacco products and, therefore, tobacco use. The Theory of Psychological Reactance posits that the perception of a threat to one’s freedom to act or not act produces an experience of reactance that motivates the individual to regain their threatened or lost freedom. There is evidence that campaigns that portray the tobacco industry as manipulating the public for their own gain may arouse psychological reactance against the tobacco industry and its products.^76^

A minority of SGM-tailored tobacco interventions (3 out of 12) referenced frameworks that more explicitly engage with the social contexts of SGM people. Fallin et al,^77^ Plant et al,^78^ and Beckerley et al^71^ described local social branding or social marketing campaigns. These were community-level approaches to behavior change based on the Diffusion of Innovations Theory.^79^ This theory focuses on the importance of social networks in the spread, maintenance, and demise of behaviors and attitudes within a population and emphasizes the role of certain influential group members in promoting or discouraging the uptake of the behavior or attitudes within the rest of the group. These interventions used commercial marketing strategies that were culturally tailored to SGM to try to shift pro-tobacco norms within SGM social networks and spaces by using anti-tobacco messaging, branded events in lesbian and gay bars, and recruitment of influential SGM community members to endorse the intervention’s anti-tobacco message.

Matthews et al^55^ work describing an SGM-tailored intervention was unique in that its conceptual framework explicitly integrated individual-level as well as SGM-specific cultural and psychosocial factors that may influence tobacco use and tobacco cessation outcomes for SGM people. Individual-level factors were drawn from the Health Belief Model and the Transtheoretical Model, while interpersonal and community-level factors were derived from the Minority Stress Model.^80^ This model explains how stigma, prejudice, and discrimination create a hostile and stressful social environment, which in turn causes mental health problems and maladaptive coping behaviors, like tobacco use, for SGM individuals.^80^ This intervention’s conceptual framework integrated predictors of cessation that are both generic as well as those that are specific to SGM groups. “Individually-mediated predictors of cessation” included perceived benefits of cessation, self-efficacy for quitting, and stage of readiness. “Cultural factors” were also accounted for, such as identification with SGM community, as well as “psychosocial factors”, namely, general stress (eg, number of stressful life events) and minority-specific stress (eg, internalized homophobia). In practice, these factors were addressed in group counseling sessions by discussing SGM determinants of tobacco use (eg, SGM social norms, SGM industry targeting) and SGM health and weight concerns. Finally, while Caldwell et al^81^ practice note did not provide an explicit theoretical basis for the intervention, it described a contextually engaged approach to a smoking cessation messaging campaign in Western North Carolina. The digital approach used cell phone locations and marketing profiles to deliver tailored smoking cessation messages to LB women while they were in the vicinity of a location known to be frequented by LB women in the area.

It would be ideal to assess the relative success of different theoretical approaches across SGM-tailored tobacco interventions, however, we found that the current body of peer-reviewed intervention outcomes is difficult to compare. Intervention delivery approaches have been heterogeneous, including in-person group sessions,^54,59^ online interventions,^56,63^ video ad campaigns, outreach letters,^66^ and hybrid digital, venue, and event-based social marketing campaigns.^77,78^ Publications reported a broad range of evaluation designs from cross-sectional pre- and post-intervention surveys within selected geographical areas^67,68,77,78^ to assessment of within-subject outcomes post-intervention,^49–51,54–56,58,59^ qualitative assessment of intervention acceptability,^66,70^ and use of single arm trials^49,50,53,59,63^ versus those with comparison and/or control groups.^53,56,58,67,68,82^

Four interventions’ effects were evaluated with randomized control or comparison group design. These likely offer^53^ the most reliable insights into theory-informed SGM-tailored tobacco intervention effectiveness to date.^53,55,56,58,67,68^ These were: (1) an SGM-tailored version of Courage to Quit,^55,58^ a group-based smoking cessation intervention delivered in SGM-serving health care centers that drew from the Transtheoretical Model, the Health Belief Model, and the Minority Stress Model, (2) the Put It Out Project,^82^ a group-based smoking cessation intervention delivered on social media that drew from the Transtheoretical Model, (3) This Free Life,^67,68^ a national digital ad campaign designed to promote anti-tobacco norms and behaviors that drew from the Theory of Psychological Reactance, the Theory of Reasoned Action, and Social Inoculation Theory,^82^ and (4) an SGM-tailored version of the Truth Campaign,^53^ a national anti-tobacco industry video ad campaign designed to promote anti-tobacco norms and behaviors, which also drew from the Theory of Psychological Reactance, the Theory of Reasoned Action, and Social Inoculation Theory. Outcomes from these four interventions showed mixed results, and are summarized below.

The prospective randomized design used to evaluate the SGM-tailored version of Courage to Quit against a non-tailored version of Courage to Quit^55,58^ found no differences between treatment groups in the primary outcome of biochemically verified smoking quit rates or in secondary outcomes but did find that the tailored version was more highly rated on acceptability than the non-tailored version (ie, program effectiveness, intervention techniques, treatment manual, being targeted to the needs of SGM individuals who smoke).

The Put It Out Project’s^56^ primary outcome, biochemically verified smoking abstinence, did not differ significantly between the randomized tailored and non-tailored Facebook treatment groups. However, secondary outcomes did show that the group receiving the tailored intervention was more likely than the non-tailored group to self-report smoking abstinence at 3- and 6 months and was more likely to report reduced smoking at 3 months (but not at 6 months). No difference between tailored and non-tailored groups was observed with regards to making a quit attempt during treatment or movement between the Transtheoretical Model’s stages of change (ie, pre-contemplation, contemplation, preparation, and action or maintenance).

Counter-industry tobacco advertisements from the Truth Campaign were tailored to target either SGM individuals or black individuals.^53^ These were evaluated with a web-based, between-subjects experimental design in which participants were randomized to watch different types of ads.^53^ The evaluation concluded that there was little evidence that the targeted counter-industry ads were especially influential in increasing support for tobacco control policies or counter-industry beliefs for either of their respective intended groups, but that the ads may have evoked anger among participants toward the tobacco industry regardless of the audience targeted.

Finally, the final evaluation of his Free Life,^67^ a primarily digital campaign designed to change tobacco-related beliefs among SGM young adults, compared end-of-campaign awareness, receptivity to, and effect of campaign exposure on tobacco-related beliefs in 24 treatment and control markets. The evaluation concluded that the campaign had modest overall effects, with high campaign awareness and receptivity but only a small effect on beliefs involving social aspects of smoking.^56^

## Discussion

Overall, individual-level theories of behavior change have dominated the design and implementation of SGM-tailored tobacco interventions as described in the peer-reviewed literature. These frameworks have value for addressing important within-person factors related to SGM-tailored tobacco interventions. For example, the focus on strengthening generalizable emotion regulation skills via acceptance and commitment therapy may be particularly relevant to supporting tobacco cessation for socially minoritized groups because it can increase resilience to minority stressors that help drive high SGM tobacco use rates.^63^ However, individual-level theories may not adequately engage with drivers of SGM tobacco use that reside within the person-environment relationship. As exceptions, some engagement has been made with the role of social networks in SGM tobacco use and cessation, as well as social minority stressors unique to SGM people that may exacerbate tobacco use and impede cessation. Building on Matthews et al. explicit integration of individual-level theories of behavior change with the Minority Stress Model,^55^ we advocate for more utilization of theoretical frameworks that account for multi-level factors relevant to SGM-tailored tobacco prevention and cessation.

There is increasing acknowledgment of the need for greater engagement with and more sophisticated conceptualizations of “context” in health behavior and health equity research,^83,84^ including tobacco-related research.^3,85–93^ Socio-ecological frameworks have been employed in tobacco use inequities research and tobacco control efforts,^3,91–93^ but this scoping review found that they have yet to be used to explicitly inform tobacco prevention and cessation intervention approaches for SGM people. The socio-ecological model has long accounted for five levels of influence on health behavior: Intrapersonal, interpersonal (including dyadic-level influences), organizational, community, and public policy.^45^ The social context of health promotion is known to impact the target health behavior as well as how health interventions are received, play out, and make an impact.^94^ For example, Williams et al.^95^ reflected on a failed attempt to implement a San Francisco-based SGM-tailored tobacco intervention in SGM communities in South Central Texas, reporting the need to address local social, cultural, and political factors when adapting and implementing existing interventions for different regions.

From a theoretical perspective, thoroughly taking context into account would mean moving beyond the typical social psychological conceptualization of “context” as the individual’s immediate internal state and location in space and time (ie, “situational triggers” of behavior) to understanding context through disciplinary lenses like those of anthropology, sociology, and human and cultural geography. These disciplinary approaches to context are underutilized in public health and health promotion research and account for the relational, dynamic, and transformative interactions between individuals and their everyday contexts. They consider social relations that are embedded in the rules, values, and resources of social structures and contexts,^83^ as well as the importance of neighborhood and ecological factors on health behavior^96^ that are determined by place-based practices and place-based regulation.^86^

A reorientation toward context in SGM-tailored tobacco intervention design, implementation, and evaluation can stimulate context-related questions that may be important for tobacco prevention and cessation outcomes. For example, how do area-level characteristics where SGM people live, such as levels of structural stigma which vary by state,^97^ interplay with how SGM people experience smoking and quitting? And, how can these contextual factors be addressed in an intervention approach to support SGM people’s smoking cessation experiences and outcomes? What additional pathways for intervention are available within place-based regulation and practices? How can public health efforts better support and reward SGM organizational policies that reject tobacco industry co-optation of LGBTQ+ Pride celebration spaces and run more tobacco-free Pride events?^98^ How can tobacco interventions influence policies and practices in nightlife spaces where SGM people have long been targeted by tobacco industry marketing and tobacco use practices are prevalent?^26^ Importantly, how can SGM-tailored tobacco interventions be designed and implemented in partnership with SGM communities such that SGM individuals are involved in multi-level efforts to support health equity and well-being in their own communities?

Empowerment Theory is one approach that may be especially effective in multi-level, community-engaged control efforts among socially minoritized groups, like SGM people, because it links individual well-being to the larger social and political environment.^99^ Empowerment Theory-informed approaches have not been used in SGM-tailored tobacco interventions, but have been found to be feasible, acceptable, and efficacious among SGM communities for HIV and STI prevention^100,101^ and are increasingly used in youth-tailored tobacco interventions.^102–104^ At the individual level, efforts to exert control are central to empowerment; empowering processes often involve participation with others to achieve goals, efforts to gain access to resources, and opportunities to gain a critical understanding of one’s sociopolitical environment.^99,105^ Individual empowerment outcomes may include perceptions of personal control, a proactive approach to life, and a critical understanding of the sociopolitical environment.^106^

For SGM individuals, SGM-related activism, and community-building participation are linked to empowering outcomes, including experiences of more meaning in life, greater community connectedness (sense of community caring), coping resources that buffer negative consequences of minority stress, and overall positive psychological functioning.^107^ Minority Stress-guided^80^ research on SGM people shows that as levels of coping and social support increase, so does engagement in health-promoting lifestyles and decreased health risk behaviors.^108^ For SGM individuals who smoke, adaptive coping strategies,^109,110^ social support,^110^ smoking abstinence self-efficacy,^111^ and internalized SGM stigma^112^ may influence tobacco use and cessation behaviors, suggesting that empowerment activities may indirectly support tobacco cessation among SGM. When integrated with individual-level behavioral frameworks, such as those outlined above, empowering approaches to SGM-tailored tobacco interventions may be particularly effective in places with high levels of SGM stigma that exacerbate tobacco and other substance use inequities among SGM people.^9^ Hatzenbuehler and Pachankis, in particular, documented the impact of SGM stigma operating at multiple levels (eg, individual, structural) on SGM individuals’ tobacco and other substance use.^4,5,9,97,113,114^ For example, the presence or absence of state-level policies that provide equal opportunities for heterosexual and sexual minority individuals and the valence of state-aggregated attitudes toward sexual minority people interacts with gay-related rejection sensitivity to predict tobacco and alcohol use for young sexual minority men.^9^

Tobacco intervention efforts that aim to address tobacco-related inequities for SGM people are building momentum. Now is an opportune time to take stock and reflect on the constraints and capacities offered by the frameworks used thus far to design interventions, and to consider integrating the strengths of individual-level behavior change frameworks with those that can explain the phenomenon of tobacco use as situated within the social, political, and physical environments of minoritized people. This can lay the foundation for an empowerment-focused intervention—an approach that has not yet been developed or evaluated for SGM tobacco cessation, despite its adoption as an effective means of promoting health equity in other areas.^100–103,115–119^ Considering the limited success of previous SGM-tailored treatments that have been largely grounded in individual-level theories of behavior change, a foundational shift may be what is needed to move the field forward.

A final observation from these scoping review findings is the lack of focused targeting of nicotine-containing products other than combustible cigarettes in SGM-tailored tobacco interventions. SGM groups have high rates of poly-tobacco product use as well as dual use of nicotine-containing products with other substances (eg, cannabis, alcohol).^120–124^ Future intervention development should target multiple substance use among SGM groups and utilize frameworks that offer well-defined conceptualizations of multiple substance use practices.^90,125–127^

There are some limitations to the current scoping review. This review did not include non-peer-reviewed (gray) literature on SGM-tailored tobacco interventions which may include theoretical underpinnings not described in the peer-reviewed literature, nor did it provide a meta-analysis of intervention design and outcomes. As a larger number of robustly validated SGM-tailored intervention outcomes are reported in the peer-reviewed literature (eg, randomized control trials) future meta-analysis of intervention outcomes because of the low number of studies that would provide data suitable for such an analysis. This should include an assessment of the degree to which focusing on broader contextual-level factors rather than individual-level factors could be more effective for SGM tobacco prevention and cessation.

## Conclusion

To conclude, at least two lessons may be gleaned from this scoping review of SGM-tailored tobacco prevention and cessation interventions: (1) The thinking that informs culturally tailored tobacco interventions for SGM people and other minoritized groups is currently driven almost exclusively by individual-level theories and (2) existing SGM-tailored interventions have had limited success in improving outcomes over non-tailored interventions. Future work to understand tobacco use inequities as indivisible from place, context, and policy may be useful for improving prevention and cessation outcomes. Working in partnership with SGM communities to address environmentally rooted drivers of SGM tobacco inequities, especially in places with high levels of SGM stigma, may be a way to enact positive change at the level of SGM individuals as well as their social and physical contexts.

## Supplementary Material

A Contributorship Form detailing each author’s specific involvement with this content, as well as any supplementary data, are available online at https://academic.oup.com/ntr.

ntad018_suppl_Supplementary_Appendix_A

ntad018_suppl_Supplementary_Appendix_B
