# Supplementary material for: Tobacco Cessation and Prevention Interventions for Sexual and/or Gender Minority-Identified People and the Theories That Underpin Them: A Scoping Review
Source: Nicotine Tob Res. 2023 Jan 31;25(6):1065–73. doi: 10.1093/ntr/ntad018 (PMC10305739; doi:10.1093/ntr/ntad018)
Supplement: ntad018_suppl_Supplementary_Appendix_A [file ntad018_suppl_supplementary_appendix_a.docx]

## Appendix A: Full Electronic Search Strategy in Ovid MEDLINE

**Search Executed:** October 28, 2022

**Search Sets Included for Review:** #01 to #23

**Database:** Ovid MEDLINE® and Epub Ahead of Print, In-Process, In-Data-Review & Other Non-Indexed Citations and Daily <January 1, 1946 to October 27, 2022>

**Search Strategy [Set; Search statement (Results)]:**

01 exp "Sexual and Gender Minorities"/ (14030)

02 (intersex$ or inter-sex$).mp. (3803)

03 (trans or transgender$ or transsex$ or transperson$ or transpeople$ or transman$ or transmen$ or transmale$ or transwom$ or transfemale$ or two spirit$).mp. (204661)

04 exp Homosexuality/ (33763)

05 Bisexuality/ (4793)

06 (lesbian$ or gay or gays or homosexual$ or bisexual$ or glbt$ or lbg$ or lgbt$ or lesbigay$).mp. (49652)

07 ((sex$ or gender$) adj2 (minorit$ or dissident$)).mp. (12315)

08 sgm.mp. (939)

09 (queer$ or non-heterosexual$ or nonheterosexual$).mp. (2773)

10 ("women who have sex with women" or wsw).mp. (292)

11 ("men who have sex with men" or msm).mp. (17585)

12 or/1-11 (261351)

13 Smoking Cessation/ (32017)

14 "Tobacco Use Cessation"/ (1409)

15 Smoking Reduction/ (104)

16 Smoking Prevention/ (18555)

17 exp Smoking/pc (607)

18 ((smoke$ or smoking$ or tobacco$ or cigar$) adj2 (cessation$ or ceas$ or stop$ or quit$ or giv$ up$ or discontin$)).mp. (52554)

19 (this free life$ or queer$ quit$ or last drag or project exhale$ or "put it out project$").mp. (7548)

20 or/13-19 (70740)

21 12 and 20 (380)

22 ..l/ 21 lg=en (368)

**23 remove duplicates from 22 (367)**
