# Supplementary material for: Tobacco Cessation and Prevention Interventions for Sexual and/or Gender Minority-Identified People and the Theories That Underpin Them: A Scoping Review
Source: Nicotine Tob Res. 2023 Jan 31;25(6):1065–73. doi: 10.1093/ntr/ntad018 (PMC10305739; doi:10.1093/ntr/ntad018)
Supplement: ntad018_suppl_Supplementary_Appendix_B [file ntad018_suppl_supplementary_appendix_b.docx]

## Appendix B: References 100-127

100. Eke AN, Johnson WD, O'Leary A, et al. Effect of a Community-Level HIV Prevention Intervention on Psychosocial Determinants of HIV Risk Behaviors among Young Black Men Who Have Sex with Men (YBMSM). *AIDS Behav* 2019;23(9):2361-74. doi: 10.1007/s10461-019-02499-4 [published Online First: 2019/04/25]

101. Kegeles SM, Hays RB, Coates TJ. The Mpowerment Project: a community-level HIV prevention intervention for young gay men. *Am J Public Health* 1996;86(8_Pt_1):1129-36. doi: 10.2105/AJPH.86.8_Pt_1.1129

102. Park E, Chang Y-P. Using Digital Media to Empower Adolescents in Smoking Prevention: Mixed Methods Study. *JMIR Pediatr Parent* 2020;3(1):e13031. doi: 10.2196/13031

103. Holden DJ, Crankshaw E, Nimsch C, et al. Quantifying the Impact of Participation in Local Tobacco Control Groups on the Psychological Empowerment of Involved Youth. *Health Educ Behav* 2004;31(5):615-28. doi: 10.1177/1090198104268678

104. Winkleby MA, Feighery E, Dunn M, et al. Effects of an advocacy intervention to reduce smoking among teenagers. *Arch Pediatr Adolesc Med* 2004;158(3):269-75. doi: 10.1001/archpedi.158.3.269

105. Zimmerman MA. Empowerment theory. Handbook of community psychology: Springer 2000:43-63.

106. Zimmerman MA. Psychological empowerment: Issues and illustrations. *Am J Community Psychol* 1995;23(5):581-99. doi: 10.1007/BF02506983

107. Szymanski DM, Goates JD, Strauss Swanson C. LGBQ activism and positive psychological functioning: The roles of meaning, community connection, and coping. *Psychol Sex Orientat Gend* 2021. doi: 10.1037/sgd0000499

108. Flenar DJ, Tucker CM, Williams JL. Sexual minority stress, coping, and physical health indicators. *J Clin Psychol Med Settings* 2017;24(3):223-33.

109. Rabois D, Haaga DAF. The influence of cognitive coping and mood on smokers' self-efficacy and temptation. *Addict Behav* 2003;28(3):561-73. doi: https://doi.org/10.1016/S0306-4603(01)00249-0

110. Rosario M, Schrimshaw EW, Hunter J. Cigarette Smoking as a Coping Strategy: Negative Implications for Subsequent Psychological Distress Among Lesbian, Gay, and Bisexual Youths. *J Pediatr Psychol* 2011;36(7):731-42. doi: 10.1093/jpepsy/jsp141

111. Gwaltney CJ, Shiffman S, Sayette MA. Situational correlates of abstinence self-efficacy. *J Abnorm Psychol* 2005;114(4):649-60. doi: 10.1037/0021-843X.114.4.649

112. Bauermeister JA, Youatt EJ, Pingel ES, et al. Psychosocial Obstacles to Smoking Cessation Attempts Among Young Adult Sexual Minority Women Who Smoke. *Behav Med* 2018;44(2):123-30. doi: 10.1080/08964289.2017.1282853

113. Hatzenbuehler ML. Structural Stigma and the Health of Lesbian, Gay, and Bisexual Populations. *Curr Dir Psychol Sci* 2014;23(2):127-32. doi: 10.1177/0963721414523775

114. Hatzenbuehler ML, Jun H-J, Corliss HL, et al. Structural stigma and sexual orientation disparities in adolescent drug use. *Addict Behav* 2015;46:14-18. doi: 10.1016/j.addbeh.2015.02.017

115. Holden DJ, Evans WD, Hinnant LW, et al. Modeling Psychological Empowerment Among Youth Involved in Local Tobacco Control Efforts. *Health Educ Behav* 2005;32(2):264-78. doi: 10.1177/1090198104272336

116. Holden DJ, Messeri P, Evans WD, et al. Conceptualizing Youth Empowerment within Tobacco Control. *Health Educ Behav* 2004;31(5):548-63. doi: 10.1177/1090198104268545

117. Mohamed H, Al-Lenjawi B, Amuna P, et al. Culturally sensitive patient-centred educational programme for self-management of type 2 diabetes: a randomized controlled trial. *Prim Care Diabetes* 2013;7(3):199-206.

118. Hahm HC, Zhou L, Lee C, et al. Feasibility, preliminary efficacy, and safety of a randomized clinical trial for Asian Women's Action for Resilience and Empowerment (AWARE) intervention. *Am J Orthopsychiatry* 2019;89(4):462-74. doi: 10.1037/ort0000383 [published Online First: 2019/07/16]

119. Kim S, Crutchfield C, Williams C, et al. Toward a new paradigm in substance abuse and other problem behavior prevention for youth: Youth development and empowerment approach. *J Drug Educ* 1998;28(1):1-17. doi: 10.2190/5et9-x1c2-q17b-2g6d

120. Nguyen N, McQuoid J, Neilands TB, et al. Same-day use of cigarettes, alcohol, and cannabis among sexual minority and heterosexual young adult smokers. *Psychol Addict Behav* 2021;35(2):215-23. doi: 10.1037/adb0000678 [published Online First: 2020/08/18]

121. Dermody SS. Risk of polysubstance use among sexual minority and heterosexual youth. *Drug Alcohol Depend* 2018;192:38-44. doi: 10.1016/j.drugalcdep.2018.07.030

122. Dermody SS, Marshal MP, Cheong J, et al. Adolescent Sexual Minority Girls Are at Elevated Risk for Use of Multiple Substances. *Subst Use Misuse* 2016;51(5):574-85. doi: 10.3109/10826084.2015.1126743

123. Hequembourg AL, Blayney JA, Bostwick W, et al. Concurrent daily alcohol and tobacco use among sexual minority and heterosexual women. *Subst Use Misuse* 2020;55(1):66-78. doi: 10.1080/10826084.2019.1656252

124. Kecojevic A, Jun H-J, Reisner SL, et al. Concurrent polysubstance use in a longitudinal study of US youth: associations with sexual orientation. *Addiction* 2017;112(4):614-24. doi: https://doi.org/10.1111/add.13681

125. Hakkarainen P, O’Gorman A, Lamy F, et al. (Re)conceptualizing “polydrug use”: Capturing the complexity of combining substances. *Contemp Drug Prob* 2019;46(4):400-17. doi: 10.1177/0091450919884739

126. McQuoid J, Keamy-Minor E, Ling P. A Practice Theory Approach to Understanding Poly-Tobacco Use in the United States. *Crit Public Health* 2020;30(2):204-19. doi: 10.1080/09581596.2018.1541226 [published Online First: 2020/09/29]

127. Kataja K, Tigerstedt C, Hakkarainen P. More social research into polydrug use. *Nord Stud Alcohol Drugs* 2018;35(6):399-403. doi: 10.1177/1455072518807055
